# Transurethral resection of ejaculatory duct combined with seminal vesiculoscopy for management of persistent or recurrent hemospermia in men with ejaculatory duct obstruction

**DOI:** 10.1186/s12894-020-00589-3

**Published:** 2020-03-23

**Authors:** Zheng-Ju Ren, Bo Yang, Dong-liang Lu, Sheng-Zhuo Liu, Lu-Chen Yang, Lin Cun Wang, Zhu-Feng Peng, Liang-Ren Liu, Qiang Dong

**Affiliations:** Department of Urology, Institute of Urology, West China Hospital, Sichuan University, 37, Guo Xue Road, Chengdu, 610041 Sichuan Province China

**Keywords:** TURED, Seminal vesiculoscopy, Haemospermia, Ejaculatory duct obstruction

## Abstract

**Background:**

Persistent or recurrent haemospermia often occurs in individuals with ejaculatory duct obstruction (EDO). This study aimed to evaluate the efficacy and safety of transurethral resection of the ejaculatory duct (TURED) combined with seminal vesiculoscopy in treating persistent or recurrent haemospermia in men with EDO.

**Methods:**

From June 2014 to March 2018, 103 consecutive patients with EDO who underwent TURED combined with seminal vesiculoscopy for persistent or recurrent haemospermia at the Department of Urology of West China Hospital were enrolled into this retrospective study. The patients were evaluated mainly by detailed history-taking and performing semen analysis, transrectal ultrasonography, and magnetic resonance imaging.

**Results:**

Among the 103 patients, 79 (76.70%) had cysts of the lower male genitourinary tract; 63 (61.17%) had blood clots; and 32 (31.07%) had calculi in the seminal vesicle and/or prostatic utricle. The duration of postoperative follow-up was 12 months, and the symptoms of haemospermia disappeared in 96 (93.20%) patients. There was no significant difference in the semen PH and sperm count before and after surgery; however, the ejaculate volume and sperm motility significantly improved postoperatively. Except for two cases of acute urinary retention and one case of watery ejaculate after surgery, no severe postoperative complications, including epididymitis, urethral stricture, urinary incontinence, retrograde ejaculation, or rectal injury, were observed.

**Conclusion:**

TURED combined with seminal vesiculoscopy is a suitable method for the diagnosis and treatment of persistent or recurrent haemospermia in men with EDO.

Haemospermia, or haematospermia, is defined by the presence of blood in the semen [1, 2]. Haemospermia has been considered as a benign and self-limiting symptom; however, it often invokes considerable anxiety and is frightening to the patient [3, 4]. It is believed that inflammation, infection, lithiasis, cyst, and obstruction in the distal seminal tract could all cause haemospermia [1, 5]. Ejaculatory duct obstruction (EDO) is a surgically correctable condition that occurs in some patients with infertility or haemospermia [6, 7], which can be sub-classified into congenital or acquired EDO. The congenital type could be caused by atresia or stenosis of the ejaculatory ducts as well as utricular, Müllerian, and Wolffian duct cysts. Conversely, the acquired type may be secondary to inflammatory or traumatic origin, including stone or post-inflammatory scar tissue formation [8, 9]. Previous studies have shown that EDO was common in patients with persistent or recurrent haemospermia [3, 10, 11]. The relationship between obstruction, inflammation, and calculus formation is vicious and can eventually lead to persistent and recurrent haemospermia [12].

Traditionally, EDO has been treated with transurethral resection of the ejaculatory duct [13, 14]. However, this surgical procedure could not remove the blood clots and calculi in the seminal vesicle or prostatic utricle for patients with persistent or recurrent haemospermia. Transurethral seminal vesiculoscopy is a common treatment for seminal tract calculi and recurrent haemospermia [15, 16]. Herein, we report our experience in using a combination of TURED and seminal vesiculoscopy to resolve persistent or recurrent haemospermia in patients with EDO.

## Methods

### Patient selection and evaluation

From June 2014 to March 2018, 103 patients with haemospermia diagnosed with EDO were treated with TURED combined with seminal vesiculoscopy in our urology centre. All patients experienced persistent or recurrent haemospermia for at least 6 months and did not improve with a course of empirical oral antibiotics during a period of 4 weeks.

**Fig. 1:**
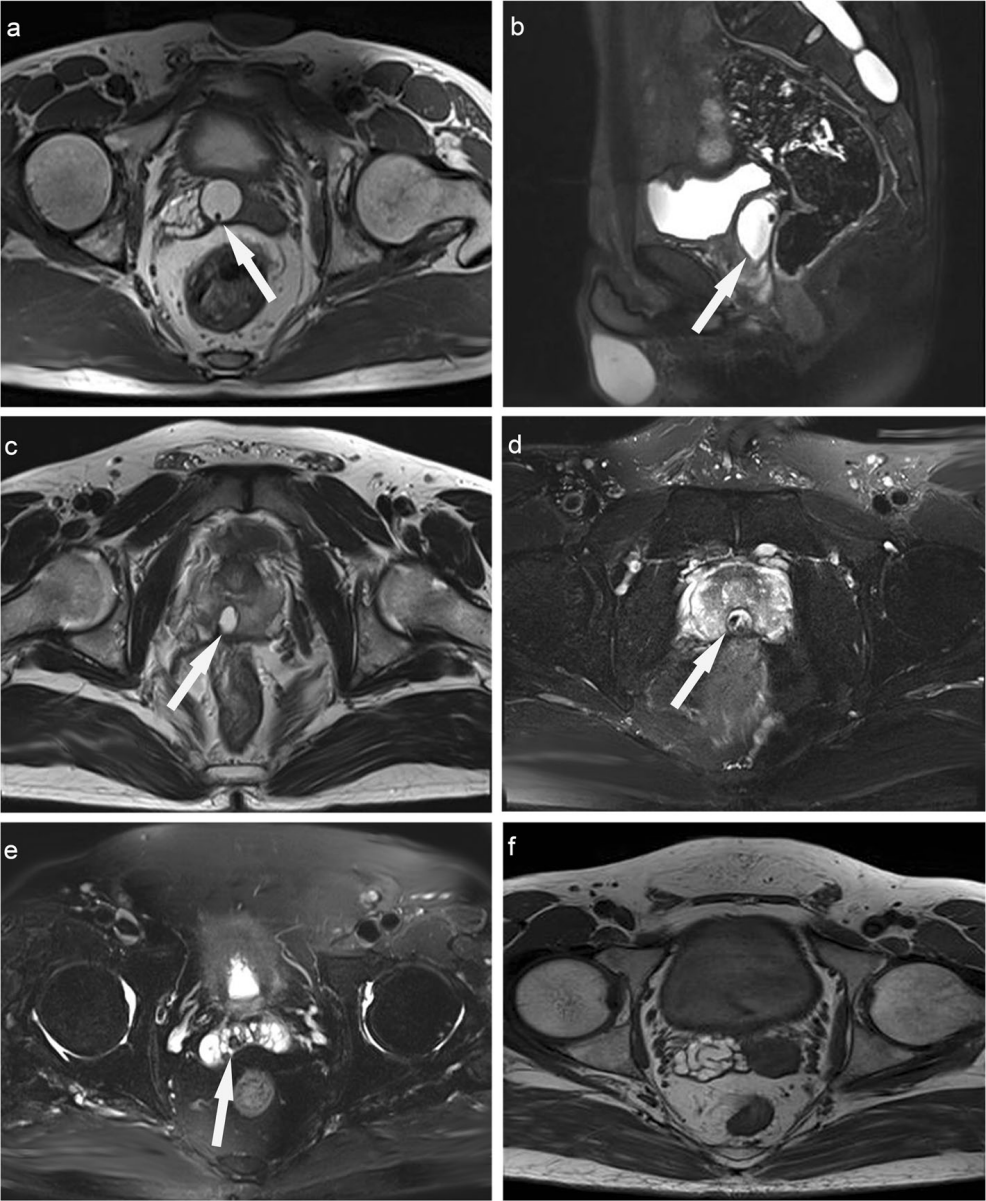
**a, b**: Prostatic müllerian duct cyst (arrow). **a**: Old haemorrhage was present in the right seminal vesicle. **c**: Ejaculatory duct cyst (arrow). **d**: Calculi were present in the prostatic utricle cyst (arrow). **e**: Calculi were present in the right seminal vesicle (arrow). **f**: old hemorrhage was present in the right dilated seminal vesicle

All relevant data including detailed history, complete physical examination, hormone profiles (FSH, LH, PRL, E2, and testosterone levels), semen analysis, TRUS and MRI were collected and analyzed. All the above relevant data of each patient were collected. In addtion, the PSA value of the middle-aged or older patients was also acquired. Semen analysis was performed at least twice for each patient. Semen samples were collected and evaluated in accordance with the World Health Organisation guidelines. EDO was confirmed by at least one of the four following findings on TRUS: (1) transverse diameter of the seminal vesicle greater than 15 mm; (2) dilated ejaculatory duct greater than 2.3 mm; (3) calcification or calculi in the ejaculatory duct and/or verumontanum; and (4) presence of a midline or eccentric cyst near the verumontanum [14, 17, 18]. MRI was used to investigate the patients with haemospermia and detected abnormalities, including cysts of the lower male genitourinary tract (Fig. 1). Semen analysis was performed preoperatively and 3 months after surgery on 90 out of the 103 patients.

### Surgical technique

**Fig. 2:**
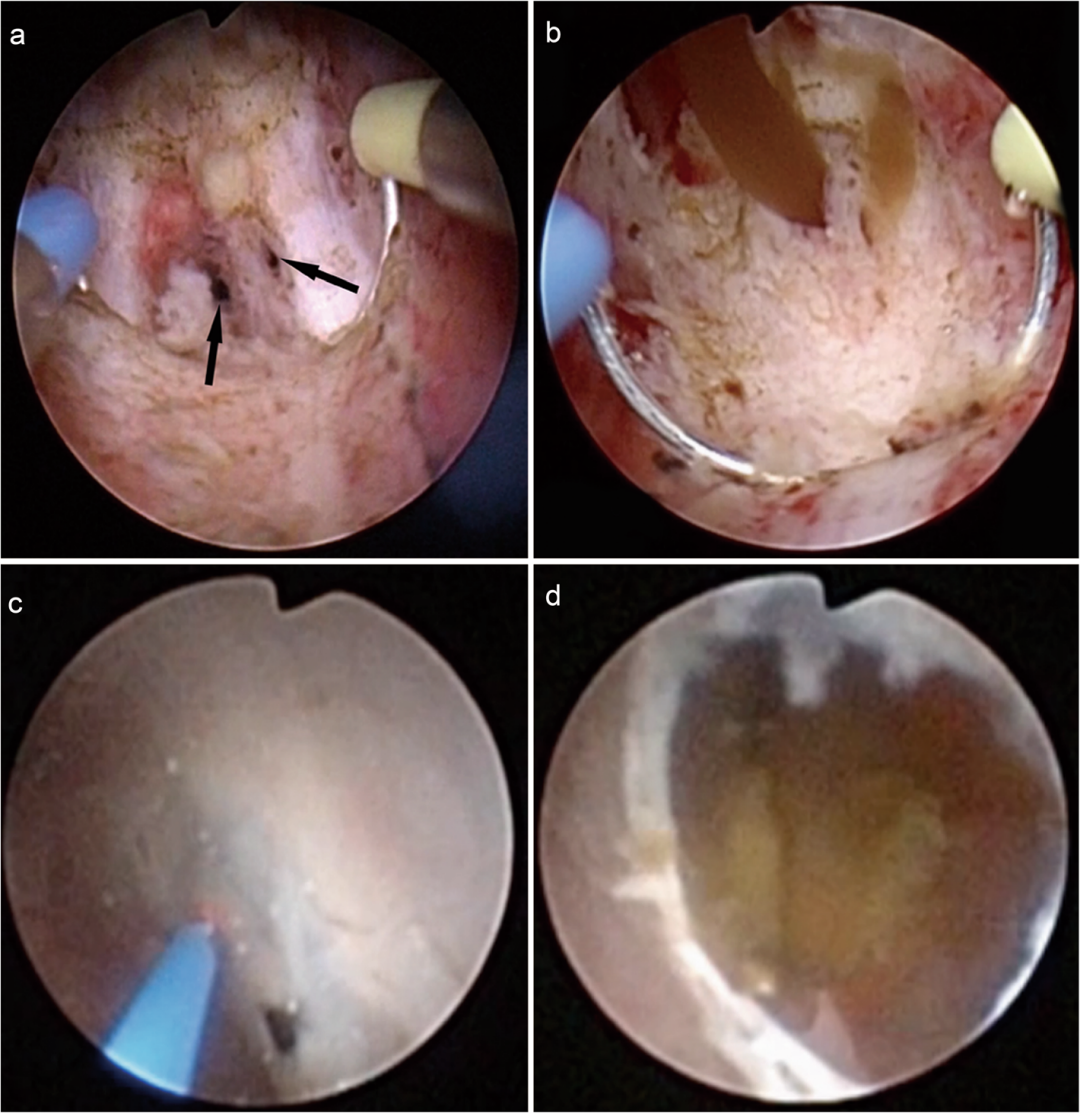
**a**: Ejaculatory ducts (arrow). **b**: Cloudy liquid flowed through the openings of ejaculatory ducts when the prostate was massaged. **c**: A direct communication fenestration was made at the thin, transparent membrane between the posterolateral wall of the prostate utricle and the seminal vesicle with holmium laser. **d**: Calculi were observed in the seminal vesicle after the communication fenestration was made

**Fig. 3:**
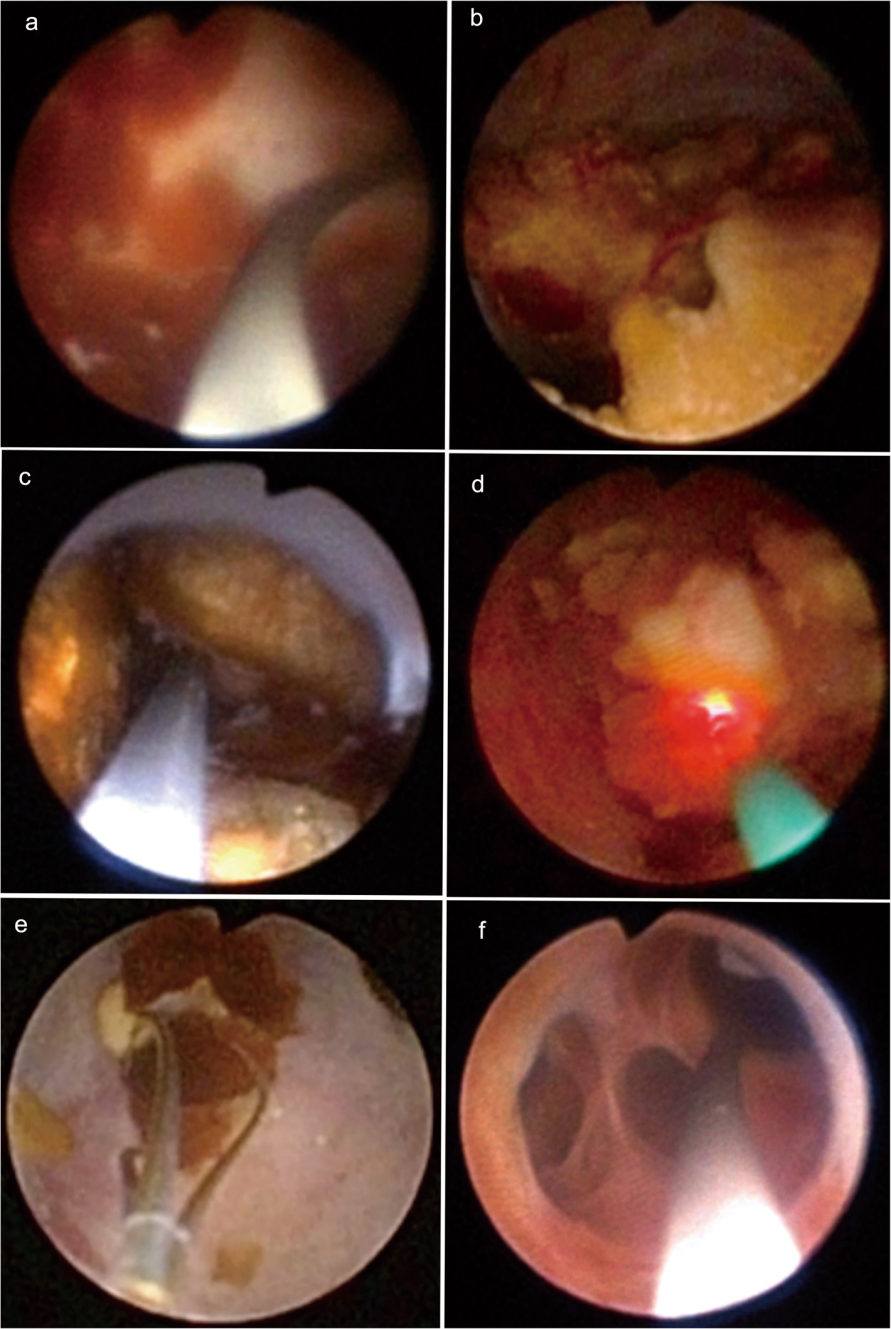
**a**: Blood clot. **b**: Calculi were present in the prostatic utricle. **c**: Calculi were present in the seminal vesicle. **d**: Calculi were fragmented with a holmium laser fiber. **e**: The fragments were removed by basket extraction. **f**: The seminal vesicle was irrigated with normal saline

Under general anaesthesia, the patients were placed in the lithotomy position. A 26-F resectoscope sheath (Olympus, Japan) was inserted into the urethra, and the resectoscope loop was used for resecting the verumontanum and ejaculatory ducts via direct dynamic video imaging (Fig. 2a). If there was a midline prostate cyst, unroofing of the cyst was performed. Directly after resection of the ejaculatory duct, the prostate was massaged to determine improved flow through the openings of the ejaculatory duct, confirming successful treatment (Fig. 2b). A flexible 6.4/7.8-Fr ureteroscope (Olympus) was inserted into the prostate utricle and the ejaculatory duct sequentially with the assistance of a guidewire, and examination or clearance of the prostate utricle and seminal vesicle was performed. If the openings of the ejaculatory duct were not found, a direct communication fenestration was created at the thin, transparent membrane between the posterolateral wall of the prostate utricle and the seminal vesicle using holmium laser (Fig. 2c-d). The blood clots or small calculi in the prostate utricle and seminal vesicle were removed directly using a ureteroscopic grasper or irrigated with normal saline. The large stones were fragmented using a holmium laser fibre under the ureteroscope, and the fragments were removed via irrigation and basket extraction (Fig. 3). After the procedure, a 16-Fr urethral Foley catheter was placed for 24 h.

### Statistical analyses

Statistical analyses were conducted using the Statistical Package for Social Sciences, version 22.0. The data in Table 1 were analysed using descriptive analyses. Paired t-tests were used to determine if there had been a statistically significant change in the semen quality after surgery. All tests were two-tailed, and the statistical significance level was set at *P* < 0.05.

**Table 1:** Demographic data (including preoperative and postoperative data)

No. of patients	103
Mean age, year (range)	40.22 (25–68)
Disease duration, months (range)	24.11 (6–120)
Follow-up duration, months (range)	13.21 (6–24)
Operation time, minutes (range)	25.27 (18–35)
Catheterization time, day	1
Hospital stay, day	1
Cysts of the lower male genitourinary tract, No. (%)	79 (76.70%)
Calculi, No. (%)	32 (31.07%)
Blood clot, No. (%)	63 (61.17%)

## Results

### Preoperative data and treatment outcomes

TURED combined with seminal vesiculoscopy was performed in 103 patients with persistent or recurrent haemospermia and EDO. The patient age ranged from 25 to 68 years (median, 40.22 years), and the mean disease duration was 24.11 months (range, 6–120 months). The mean follow-up duration was 13.21 months (range, 6–24 months). There were 28 older men who have tested PSA value, the PSA value ranged from 0.168 to 3.790 ng/ml (median, 1.834 ng/ml). Before surgery, hematocele in the seminal vesicle were detected in all patients by TRUS and MRI. The calculi were detected in 32 patients by TRUS and MRI. The physical examination findings and hormonal levels were normal in all patients. TURED combined with seminal vesiculoscopy was successfully performed in all patients. The mean surgical time and hospital stay duration were 25.27 min and 1 day, respectively. All urethral catheters were removed 1 day after surgery. Of the 103 patients, 92 patients had unilaterally EDO and 11 patients had bilateral EDO. In these patients, 32 had calculi, and 63 had blood clots in the seminal vesicle or prostate utricle. There were 19 patients had calculus in left seminal vesicle, 13 patients had calculus in right seminal vesicle. The symptoms of haemospermia disappeared in 96 of the 103 (93.20%) patients after subsequent semen analysis one to three times during the follow-up period. In addition, the symptoms of haemospermia persisted in 7 patients after subsequent semen analysis one to three times. The persisted hematospermia may due to the inflammatory and infectious. All the 7 patients were treated by oral antibiotics, and symptoms of hemospermia disappeared after 5-7 days. The demographic data of the patients are presented in Table 1.

Semen analysis was performed preoperatively and 3 months postoperatively in 90 out of the 103 patients. The semen analysis parameters, including the ejaculate volume, semen PH, sperm concentration, and percent motility, are listed in Table 2. During the postoperative period, the ejaculate volume and percent motility significantly increased, whereas the semen PH and sperm concentration did not change significantly.

**Table 2:** Semen analysis data

Semen variable	Before treatment	After treatment	P
Ejaculate volume	1.76 ± 0.69	2.06 ± 0.68	0.004
Ejaculate PH	7.22 ± 0.08	7.32 ± 0.05	0.209
Sperm count (×10^9^/ml)	62.52 ± 58.16	67.32 ± 49.16	0.060
Sperm motility (%)	59.71 ± 27.32	67.48 ± 23.32	0.020

### Complications

Three patients experienced complications. One patient complained of watery ejaculate, which may be due to urine reflux retrograde through the ejaculatory ducts and seminal vesicle. In two patients, acute urinary retention was reported; they were treated and recovered after using a urine catheter for 1 week. No severe complications, such as epididymitis, retrograde ejaculation, urinary incontinence, or rectal injury, were observed in all patients after surgery.

## Discussion

Haematospermia, or haemospermia, is usually regarded as a benign and self-limiting symptom, requiring no additional treatment or evaluation [19, 20]. However, the condition is often associated with impaired quality of life owing to induced anxiety and must be taken seriously by both patients and physicians, particularly if it is recurrent and refractory and has co-existing pain [1, 2]. In addition, patients present to their primary care physician after a single episode of haemospermia out of concern for malignancy or venereal disease [20]. Evaluating the aetiology is the best approach to the initial management of hemospermia. In the present study, 103 patients with persistent or recurrent haemospermia and EDO were treated with TURED combined with seminal vesiculoscopy. These results indicate that TURED combined with seminal vesiculoscopy is a safe and effective technique for the management of patients with refractory haemospermia and EDO.

There are many possible causes of hemospermia, most of which are benign, and the risk of malignancy is low [21, 22]. In 2013, Li analysed the pathogenesis of persistent and refractory hemospermia in 102 patients, of which 88 (86.3%) patients showed typical and characteristic changes in the ejaculatory duct area, and some degrees of EDO were found during their surgery [5]. In the recent study conducted by Chen in 2018, transurethral seminal vesiculoscopy was performed in 419 patients with persistent haemospermia in Shanghai Changhai Hospital (Shanghai, China) from May 2007 to November 2015 [11]. Ejaculatory duct stenosis or EDO, mucosal lesions in the seminal vesicle, and calculi in the seminal vesicle or verumontanum were observed in 312 (81.9%), 209 (54.9%), and 19 (5.0%) cases, respectively [11]. In our study, we enrolled 103 patients with persistent haemospermia and EDO. Cysts of the lower male genitourinary tract were found in 79 patients; calculi in the seminal vesicle or prostate utricle in 32 patients; and blood clots in 63 patients during surgery. EDO is considered as the most common cause of refractory hemospermia, and the risk of EDO might be higher in patients with hemospermia. If EDO is not resolved, the blood clots and calculi in the seminal vesicle or ejaculatory duct cannot be effectively discharged, which may lead to persistent or recurrent haemospermia. Therefore, the key point to the treatment of persistent or recurrent haemospermia in patients with EDO is early diagnosis, timely drainage, and relief of obstruction.

TURED, considered as the standard procedure for the management of EDO, was initially described by Farley and Barnes in 1973 [23]. Some studies have reported that TURED is a viable and minimally invasive option for treating EDO caused by ejaculatory disorders, including infertility and hemospermia [10, 24, 25]. In 2008, Manohar et al. investigated 25 patients with ejaculatory disorders, including hemospermia, who underwent TURED between 1997 and 2005, and all patients complained of symptoms, including painful ejaculation and hemospermia; they observed complete remission of symptoms 3 months after surgery [10]. Transurethral seminal vesiculoscopy is a new technique used for the diagnosis and treatment of seminal tract diseases. The first report of in vivo endoscopic evaluation of the seminal vesicles was provided by Yang in 2002 [26]. Recently, several reports have described the endoscopic technique for the management of patients with seminal tract diseases [16, 27]. Liu et al. investigated 72 patients with haemospermia who underwent transurethral seminal vesiculoscopy and treatment at Shanghai Changhai Hospital between 2006 and 2008 [4]. Their analysis showed that definite diagnosis was made in 93.1% of patients, and 94.4% were cured or at least reported alleviation of their symptoms. Tang et al. evaluated 30 patients with persistent hemospermia who were treated with transurethral seminal vesiculoscopy between November 2013 and January 2016 in the First Affiliated Hospital of Fujian Medical University [28]. Calculi in the ejaculatory duct or seminal vesicle were found in 20 patients, and inflammation or dark red jelly-like substances in the seminal vesicle were observed in all patients [29]. In our study, all patients were treated with TURED combined with seminal vesiculoscopy; of them, 32 had calculi in the seminal vesicle or prostate utricle, and 63 had blood clots. The symptoms of haemospermia disappeared in 96 of the 103 (93.20%) patients after surgery. In addition, the ejaculate volume and percent motility significantly increased. In patients with haemospermia and EDO, the vicious cycle of obstruction, inflammation, and calculus formation may eventually lead to recurrent and refractory hemospermia. TURED combined with transurethral seminal vesiculoscopy is a simple, minimally invasive procedure that is performed via the urogenital tract. Its use can remove obstructions, blood clots, and calculi, thereby relieving symptoms and reducing the risk of recurrence. However, although the surgical procedure is not very difficult, the potential risk of ejaculatory duct and seminal vesicle structural damage remains. Therefore, careful performance of surgical procedures is required, especially in unmarried men who plan to have children.

The present study had a number of limitations. The sample size was small owing to the low rates of persistent haemospermia with EDO. Additionally, the study was a single-arm, observational study without a control group; thus, the beneficial effects of the surgery might have been overestimated. A randomised controlled trial is required to provide reliable evidence in the future. The follow-up period in a proportion of the patients after the treatment may not be of sufficient duration; long-term follow-up results are required to clarify the efficacy and safety of this surgical procedure. Despite these limitations, to the best of our knowledge, this is the first study to show the efficacy and safety of TURED combined with seminal vesiculoscopy for the treatment of persistent or recurrent haemospermia in patients with EDO. Our analysis showed that 93.20% of the patients had completely relieved haemospermia symptoms, and the percent motility significantly increased after surgery; these results are comparable to the results of previous studies. The results of our study provide important preliminary information indicating that TURED combined with seminal vesiculoscopy could be an alternative treatment strategy for persistent or recurrent haemospermia in patients with EDO.

## Conclusion

TURED combined with seminal vesiculoscopy is an excellent alternative for persistent or recurrent haemospermia in patients with EDO who are refractory to medical treatment. The remission of symptoms and improvement of the semen quality may be mediated through the effects of the removal of obstructions, blood clots, and calculi by this surgical procedure. Additional research is needed to understand the anatomy of the distal seminal tracts and further examine the efficacy and safety of this surgery for persistent or recurrent haemospermia in patients with EDO.

## Data Availability

The datasets used and/or analysed during the current study are available from the corresponding author on reasonable request.

## Abbreviations

EDO: Ejaculatory duct obstruction
MRI: Magnetic resonance imaging
TRUS: Transrectal ultrasonography
TURED: Transurethral resection of ejaculatory duct

## References

[1] Suh Y, Gandhi J, Joshi G, Lee MY (2017). Etiologic classification, evaluation, and management of hematospermia. Transl Androl Urol.

[2] Mathers MJ, Degener S, Sperling H (2017). Hematospermia-a symptom with many possible causes. Dtsch Arztebl Int.

[3] Li YF, Liang PH, Sun ZY (2012). Imaging diagnosis, transurethral endoscopic observation, and management of 43 cases of persistent and refractory hematospermia. J Androl.

[4] Liu ZY, Sun YH, Xu CL (2009). Transurethral seminal vesiculoscopy in the diagnosis and treatment of persistent or recurrent hemospermia: a single-institution experience. Asian J Androl.

[5] Li BJ, Zhang C, Li K (2013). Clinical analysis of the characterization of magnetic resonance imaging in 102 cases of refractory haematospermia. Andrology..

[6] Jarow JP (1996). Transrectal ultrasonography in the diagnosis and management of ejaculatory duct obstruction. J Androl.

[7] Wang HF, Ye HM, Xu CL (2012). Transurethral seminal Vesiculoscopy using a 6F Vesiculoscope for ejaculatory duct obstruction: initial experience. J Androl.

[8] Fisch H, Lambert SM, Goluboff ET (2006). Management of ejaculatory duct obstruction: etiology, diagnosis, and treatment. World J Urol.

[9] McQuaid JW, Tanrikut C (2013). Ejaculatory duct obstruction: current diagnosis and treatment. Curr Urol Rep.

[10] Manohar T, Ganpule A, Desai M (2008). Transrectal ultrasound- and fluoroscopic-assisted transurethral incision of ejaculatory ducts: a problem-solving approach to nonmalignant hematospermia due to ejaculatory duct obstruction. J Endourol.

[11] Chen R, Wang L, Sheng X (2018). Transurethral seminal vesiculoscopy for recurrent hemospermia: experience from 419 cases. Asian J Androl.

[12] Xing C, Zhou X, Xin L (2012). Prospective trial comparing transrectal ultrasonography and transurethral seminal vesiculoscopy for persistent hematospermia. Int J Urol.

[13] Paick JS, Kim SH, Kim SW (2000). Ejaculatory duct obstruction in infertile men. BJU Int.

[14] El-Assmy A, El-Tholoth H, Abouelkheir RT (2012). Transurethral resection of ejaculatory duct in infertile men: outcome and predictors of success. Int Urol Nephrol.

[15] Suh Y, Gandhi J, Joshi G (2017). Etiologic classification, evaluation, and management of hematospermia. Transl Androl Urol.

[16] Kang PM, Seo WI, Yoon JH (2016). Transutricular seminal vesiculoscopy in the management of symptomatic midline cyst of the prostate. World J Urol.

[17] Purohit RS, Wu DS, Shinohara K (2004). A prospective comparison of 3 diagnostic methods to evaluate ejaculatory duct obstruction. J Urol.

[18] Orhan I, Duksal I, Onur R (2008). Technetium Tc 99m Sulphur colloid seminal vesicle scintigraphy: a novel approach for the diagnosis of the ejaculatory duct obstruction. Urology..

[19] Mittal PK, Camacho JC, Sahani DV (2016). Hematospermia Evaluation at MR Imaging. Radiographics..

[20] Leocadio DE, Stein BS (2009). Hematospermia: etiological and management considerations. Int Urol Nephrol.

[21] Stefanovic KB, Gregg PC, Soung M (2009). Evaluation and treatment of hematospermia. Am Fam Physician.

[22] Badawy AA, Abdelhafez AA, Abuzeid AM (2012). Finasteride for treatment of refractory hemospermia: prospective placebo-controlled study. Int Urol Nephrol.

[23] Farley S, Barnes R (1973). Stenosis of ejaculatory ducts treated by endoscopic resection. J Urol.

[24] Yurdakul T, Gokce G, Kilic O (2008). Transurethral resection of ejaculatory ducts in the treatment of complete ejaculatory duct obstruction. Int Urol Nephrol.

[25] Modgil V, Rai S, Ralph DJ (2016). An update on the diagnosis and management of ejaculatory duct obstruction. Nat Rev Urol.

[26] Yang SC, Rha KH, Byon SK (2002). Transutricular seminal vesiculoscopy. J Endourol.

[27] Han WK, Lee SR, Rha KH (2009). Transutricular seminal vesiculoscopy in hematospermia: technical considerations and outcomes. Urology..

[28] Tang SX, Zhou HL, Ding YL (2016). Effectiveness of transurethral seminal vesiculoscopy in the treatment of persistent hematospermia, and oligoasthenozoospermia and azoospermia from ejaculatory duct obstruction. Nat Med J China.

